# Evaluation of the Biases in the Studies that Assess the Effects of the Great Recession on Health. A Systematic Review

**DOI:** 10.3390/ijerph16142479

**Published:** 2019-07-11

**Authors:** Marc Saez, Maria Antònia Barceló, Carme Saurina, Andrés Cabrera, Antonio Daponte

**Affiliations:** 1 Research Group on Statistics, Econometrics and Health (GRECS), University of Girona, 17003 Girona, Spain; 2 Network Biomedical Research Center of Epidemiology and Public Health (CIBERESP), 28029 Madrid, Spain; 3 Andalusian School of Public Health, 18080 Granada, Spain; 4 Instituto de Investigación Biosanitaria (IBS), Hospital Universitario de Granada, Universidad de Granada, 18014 Granada, Spain; 5 Observatorio de Salud y Medio Ambiente de Andalucía (OSMAN), 18080 Granada, Spain

**Keywords:** health, great recession, bias, evaluation problem, time bias

## Abstract

Background: Our main objective was to evaluate the fundamental biases detected in studies assessing the effects the Great Recession had on health for the case of Spain. As secondary objectives we presented methods to control these biases and to discuss the results of the studies in question if they had controlled for them. Methods: We carried out a systematic review of the literature published up to June 2018. We evaluated the biases that could have happened in all the eligible studies. Results: From the review, we finally selected 53 studies. Of the studies we reviewed, 60.38% or 32 out of 53, were evaluated as having a high risk of bias. The main biases our review revealed were problems with evaluation, time bias, lack of control of unobserved confounding, and non-exogeneity when defining the onset of the Great Recession. Conclusions: The results from the studies that controlled the biases were quite consistent. Summing up, the studies reviewed found that the Great Recession increased the risk of declaring poor self-rated health and the deterioration of mental health. Both the mortality rate and the suicide rate may well have increased after the Great Recession, probably after a three- to four-year delay.

## 1. Introduction

The Great Recession was the period of general economic decline that, according to the International Monetary Fund, has been the worst global recession since the 1930s [1]. The recession took place between 2007 and 2013, albeit with some differences between countries in its timing and scale. Perhaps it could be too optimistic to say that the crisis has ended. In Spain, the unemployment rate is (in the 12th year of crisis) still equal to 14%. It is twice higher than before the crisis (7%). Approximately, there is the same situation in Italy where unemployment is equal to 10% (before crisis it was 5%), and even in France the pre-crisis unemployment rate has not been reached yet (8%, and before crisis it was 7%).

There have been a lot of studies that have assessed the effects of the Great Recession on health. For example, in our systematic search, we found 8673 studies, including 835 systematic literature reviews, among the studies published until June 2018.

However, the results of those studies are very heterogeneous and, in most of the health outcomes, also inconsistent. Parmar et al., in a recent systematic review of studies about the effects of the 2008 financial crisis on European countries, argue that this heterogeneity could be attributed to the country and group of population analyzed [2]. However, much of the heterogeneity and inconsistency in the results might well be attributed to methodological errors that could compromise the quality of the studies. 

At least until June 2018, Parmar et al. were the only ones who had conducted a risk of bias assessment of the studies they had reviewed. They allocated each study an overall rating of either ‘strong’, which is a low risk of bias if none of the seven domains they considered had been rated as weak (weak being a score equal to 3); ‘moderate’: A moderate risk of bias if up to two domains were rated as weak; or ‘weak’: A high risk of bias if three or more domains were rated as weak. As only two of the 41 studies they reviewed (i.e., 5%) rated ‘strong’ (‘low risk of bias), Parmar et al. recommend caution when interpreting the results [2]. 

Our main objective in this paper is to evaluate the main biases detected in those studies that assess the effects of the Great Recession on health for the case of Spain. As secondary objectives we intend to show the methods with which to control these biases, as well as to discuss the results of those studies had they controlled for bias. 

To achieve our objectives, we conducted a systematic review for the case of Spain. Our reasons for choosing Spain were twofold: Spain is one of the European countries in which the Great Recession was most severe and one in which it lasted longest (the eighth country in the world, the seventh in Europe, and the sixth in the Eurozone) [3]. Spain is, together with Greece, one of the countries where most studies have been focused. In the recent systematic review by Parmar et al., Spain had 10, out of a total of 41, articles dedicated to it and Greece had nine [2]. Adding ‘Spain’ as the keyword and the Boolean operator ‘AND’ to the bibliographic search outlined above, revealed 371 studies and 347 when the keyword was ‘Greece’.

The article is organized as follows. After this introduction, in Section 2, we explain the methods used for the systematic review and for the evaluation of the biases that could be found in the different studies. We explain the results in Section 3, commenting, specifically, on the problem of evaluation and time bias. We discuss our findings in Section 4 and conclude, pointing out strengths and limitations, in Section 5.

## 2. Materials and Methods

### 2.1. Systematic Review

We conducted a search in the online databases: PubMed, Web of Science, Scopus, and Google Scholar, up to June 2018, by combining the keywords ‘health’ and ‘Spain’ with the keywords ‘(economic crisis)’, ‘(economic downturn)’, ‘(economic recession)’, or ‘(financial crisis)’, through the Boolean connector ‘AND’. We included studies published in English or Spanish. We excluded qualitative studies, opinion or purely narrative papers, and systematic literature reviews. We also excluded those studies in which the response variable was not a health outcome, for example those that analyze changes in the healthcare system or provide indicators to assess the impact of the crisis on health.

**Figure 1:**
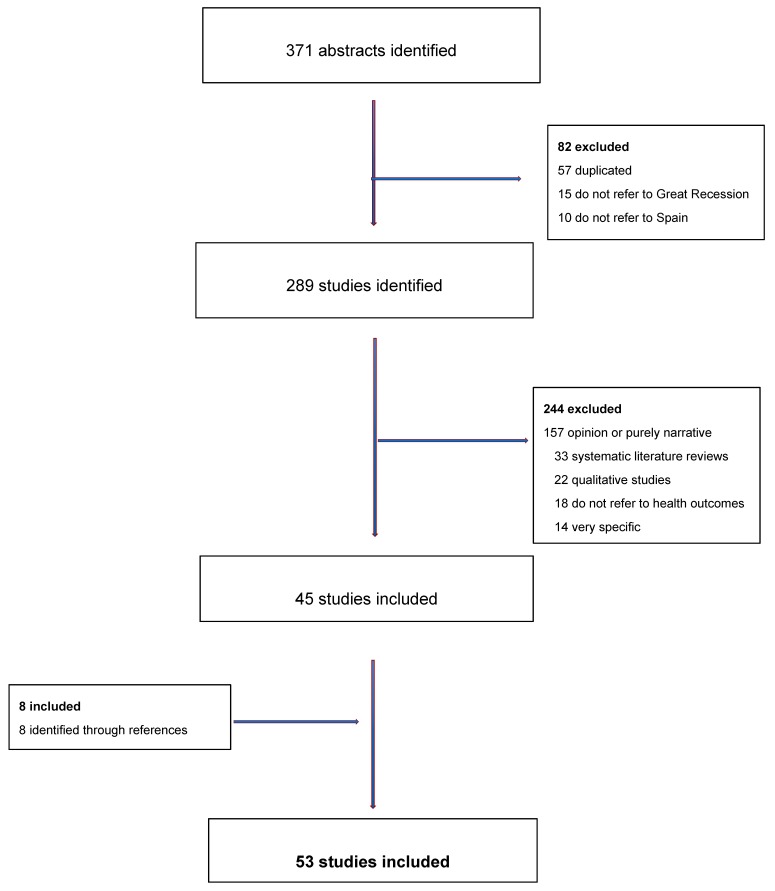
Flowchart of the selection of studies.

One of us read the titles and abstracts of the studies and made the first selection by discarding any duplicates and making a first exclusion of those studies that neither referred to Spain nor to the Great Recession. Three of us then read and discussed the full text of the remaining studies and excluded those that did not meet the inclusion criteria. Then, we checked the references and citations of the selected studies and any additional studies identified were reviewed following the same strategy. Finally, we agreed on the final list of studies to be included for analysis (Figure 1). In the review process, we followed the preferred reporting items for systematic reviews and meta-analysis (PRISMA) [4] protocols. 

### 2.2. Evaluation of Biases

In the evaluation of biases, we used six of the seven domains from the tool developed by Parmar et al. [2]: (i) Selection bias, (ii) confounding bias, and iii) measurement error in health outcome and defined three new domains: (iv) Measurement error in exposure variable, (v) ecological fallacy, and vi) time bias. Unlike Parmar et al., we did not use their dominion ‘reporting bias’. We believe that, unlike the others, the subjective aspects this domain contains hinders its use. 

We added two additional domains: ‘Evaluation problem’ and ‘unobserved confounding’. ‘Evaluation problem’ is a consequence of using observational data from non-experimental designs [5] and occurs when an event is evaluated (for example, the Great Recession) using data in the form of a cross section (for example, health surveys) before and after the event. The problem is that the subjects interviewed before and after the event are not the same ones, thus making the pre- and post-event groups not comparable. ‘Unobserved confounding’ evaluates the control that the study made of the heterogeneity and of the temporal extra variability. Heterogeneity is the presence of unobserved variables (i.e., unobserved confounding) invariant over time, that is specific to the units of analysis. Temporal extra variability refers to the existence of temporal trends that, if are not controlled for, could confound the relationships of interest (see Table 1).

In each study, each of the eight domains were rated following Parmar et al., i.e., 1—low risk of bias, 2—moderate risk of bias, and 3—high risk of bias) [2]. The two domains that we added were scored as 1 if more than one method was used to control the bias, as 2 if only one method was used, and as 3 if the bias was not controlled (Table 1). 

For the overall rating of each study, we once again followed Parmar et al., evaluating as ‘strong’ (low risk of bias) if none of the eight domains was rated as high risk of bias (i.e., a rating of 3), ‘moderate’ (moderate risk of bias) if up to two domains were rated as weak, or ‘weak’ (high risk of bias) if three or more domains were rated as high risk of bias.

Two of us assessed each study independently. Finally, three of us agreed on the rating for each domain and the overall rating of the study.

## 3. Results

### 3.1. Systematic Review

Figure 1 is a flowchart of the review process. Of the 371 abstracts initially identified, and after excluding duplicates and studies that were not related to Spain or the Great Recession, we were left with 289 studies. Of these, 45 were finally eligible. From the citations and references we included 8 more studies, which left us with a final total of 53 studies (see Table 2). The complete list of studies is shown in the supplementary file (Supplementary Table S1, studies include in the systematic revision).

The health outcomes analyzed by these studies were mental health (23 studies, 43.4%), self-rated health (16 studies, 30.2%), suicides (9 studies, 17.0%), mortality (8 studies, 15.1%), alcohol and illegal drugs (4 studies, 7.5%), use of and access to health services (4 studies, 7.5%), health inequalities (3 studies, 5.7%), and others including health-related quality of life, dental care, sexual and reproductive health, infectious diseases, life expectancy, small for gestational age, and underweight at birth (6 studies, 11.3%). Almost 36% of the studies (19 out of 53) analyzed the effects on more than one health outcome. 

In 58.5% of the studies, the leading authors’ institution (the first author and the corresponding and the senior authors) was a university and in 30.2%, of the cases a health agency. In most cases, the leading authors had more than one affiliation. Catalonia (with 34.0%), Andalusia (with 17.0%), and Madrid (with 13.2%) accounted for 64.2% of the geographical origins of the studies. It should be noted that 11.3% of the studies had their geographical origin outside of Spain.

### 3.2. Risk Assessment

Of the 53 eligible studies evaluated, 10 had already been assessed in Parmar et al. [2]. However, our evaluations differed to theirs in the ‘Selection bias’ domain in six studies. Four of these six articles received a score of 3 and so were evaluated by Parmar et al. as high risk (‘response rate <60% or not reported’; despite using the same data, with a score of 2, the other two studies were rated as moderate risk. However, all used the same health surveys at a national level (or at a regional level, in two of the cases), which have a response rate higher than 80%. Thus, we believe that, as they received a score of 1, they should in fact be rated as low risk. 

We evaluated 39.6% of the studies (21 of 53) as moderate or low risk, and only one as having low risk. We evaluated 12.5% (1 of 8) of the health outcome ‘mortality’, 20.0% (3 of 15) of ‘self-perceived health’, 25.0% (1 of 4) of ‘use of and access to health services’, 26.1% (6 of 23) of the health outcome ‘mental health’, 28.6% (2 of 7) of ‘other’, 44.4% (4 of 9) of ‘suicides’, 50.0% (2 of 4) of ‘alcohol and illegal drugs’, and 100% (3 of 3) of ‘health inequalities’ as having a low or moderate risk. 

### 3.3. Evaluation Problem

As we said above, the evaluation problem occurs when trying to assess the effects of the Great Recession on health using data in the form of a cross section (i.e., health surveys). The problem is that the subjects interviewed before and after the Great Recession are not the same and, therefore, not comparable. Health surveys were used in most of the studies that analyzed the health outcomes ‘self-perceived health’, ‘mental health’, ‘use of and access to health services’, and ‘alcohol and illegal drugs’. 

Of the 12 studies reviewed by Parmar et al. [2] that assessed the effect of the crisis on self-perceived health, five referred to Spain. Those five were precisely the only ones to conclude that the crisis decreased the probability of declaring poor health (i.e., fair, bad, or very bad self-perceived health). None of the five controlled for the evaluation problem. Nor was it controlled for by two of the studies, which also assessed the effects of the crisis on self-perceived health that we reviewed but had appeared after Parmar et al.’s study [6,32]. These two studies also found an improvement in self-perceived health during the Great Recession. Similarly, studies that assessed the effects of the crisis on mental health indicated that the risk of its deterioration decreased [11,18] (particularly for young women) or did not change (especially in men).

However, these results could be a consequence of a lack of control of the evaluation problem. The control can be done without changing the design. In the first case, the control is equivalent to controlling confounding and three strategies could be followed to do so: Matching, stratification, and adjustment in a multivariate model. 

With respect to matching, when Arroyo et al. [29] and Urbanos-Garrido and López-Valcárcel [36] evaluated the effects of the crisis on self-perceived health, they used the same data as the studies that do not control this bias (i.e., the Spanish Health Survey in both cases, and also the Catalan Health Survey in the second case, for the years 2006 and 2011–2012). Matching the subjects from the samples before and after the crisis, they found that the crisis did not alter the likelihood of reporting poor health for the general population [29], and that unemployment had a significant negative impact on self-rated health, particularly for the long-term unemployed [36]. 

In terms of stratification, some studies, most of which, besides analyzing the complete sample, evaluated specific groups of the population such as those most vulnerable (the unemployed, immigrants, individuals with a low level of education, people affected by, or at risk of, foreclosure or eviction, and/or the groups with the greatest risk of vulnerability: Children and the elderly) all found that the risk of declaring both poor health and poor mental health actually increased once stratification had been carried out. This is because the members of these groups, despite not being the same as before or after the crisis, shared some common characteristics and, therefore, were more comparable. 

A third strategy is to use a multivariate model to control explicitly for confounding. In fact, this corresponds to a domain already considered by Parmar et al. [2]. Using the Spanish National Health Surveys for 2006 and 2011–2012, Pérez-Romero et al. [21] and Arroyo et al. [7] adjusted for many confounders, among them social support variables [21] and interactions between the year of the survey and occupation, unemployment (distinguishing between short- and long-term unemployment), educational level, and the social class of the subject [7]. Pérez-Romero et al. found that the crisis did not change the probability of declaring poor health, although self-perceived mental health worsened in men [21]. Arroyo et al., found that at-risk children were the most negatively affected in the Spanish economic downturn [7]. Meanwhile, using two waves of the Andalusian Health Survey (2007 and 2011–2012), Córdoba-Doña et al., included financial strain and social support among the confounders and found that the prevalence of poor mental health increased during the crisis compared to the pre-crisis period, although differentially according to labor market status and education level [12]. In particular, those with secondary studies may have had a higher risk. They also found that financial strain could have a partial mediating role for the effects of crisis on poor mental health for the unemployed [12]. Gili et al., using data from random samples of two nationwide cross-sectional surveys (2006–2007 and 2010–2011), found that the prevalence of mental disorders increased significantly between 2006 and 2010 [14,16], especially for families experiencing unemployment and mortgage payment difficulties [14] and for men [16]. 

Gili et al. also found that the Great Recession significantly increased alcohol-related disorders [14]. However, using a multivariate model, which they adjust by using a large number of confounders, Martín-Bassols and Vall-Caselló found that there while there was a decrease in alcohol consumption, there was also an increase in abusive smoking behavior (smoking every day) and in the consumption of illegal drugs (marihuana and cocaine) [54]. Although they did not completely control the evaluation problem and caution should be exercised when considering their results, Collell et al. [53] and Bosque-Prous et al. [52], using the same data and two waves of the SHARE project, also obtained similar results. Meanwhile, Bosque-Prous et al. found that the prevalence of hazardous drinking (an average daily consumption of >2 and >3 alcoholic drinks in the previous three months in women and men, respectively) decreased in men and the proportion of abstainers (not drinking any alcoholic beverage during the three months prior to the interview) increased in men [52]. Collell et al. found that alcohol use remained stable and heavy drinking decreased, although binge drinking increased [52]. Further, they found that sporadic cannabis use increased [52]. 

Using data from the Spanish National Health Surveys 2006 and 2011–2012, a multivariate model adjusted by a large number of confounders was also used by Abásolo et al. [55], who analyzed the health-care utilization patterns of different income groups, and by García-Subirats et al. [58] who analyzed changes in access to healthcare and its determinants in the immigrant and native-born populations. Both found that the onset of the Great Recession was accompanied by a decrease in general practitioner visits [55,58] (although only among the native-born in García-Subirats et al. [58]) and hospitalizations [55], and an increase in specialist visits [55,58], while the use of emergency visits remained constant [55]. Lostao et al., who analyzed not only Spain but also Germany, found that in the case of Spain, there were no differences either in general practitioner visits or in hospitalizations [59]. However, it must be taken into account that they did not include as many confounders and, therefore, would not have completely controlled the evaluation problem. Using data from the Andalusian Health Survey 2007 (pre-crisis) and 2011–2012 (crisis), Córdoba-Doña et al. found that inequalities in healthcare utilization remained in favor of the most disadvantaged [57], although it should be noted that we evaluate this study as a high risk of bias.

The evaluation problem would not exist if a different design had been used. Specifically, a longitudinal one with repeated measurements of the same individuals before and after the crisis, so that the individual is their own control. This was the strategy followed by Zapata-Moya et al. [28], López-del-Amo et al. [34], and Fornell et al. [33], when they used the longitudinal data from the European Union Statistics on Income and Living Conditions (EU-SILC) survey. They all found that the crisis or its consequences (unemployment, job insecurity, and poverty) increased the risk of declaring poor health.

Using population-based cohorts for regions of Catalonia, Sicras-Mainar and Navarro-Artieda [26] and Barceló et al. [9] investigated the evolution of the use of psychotropics before and after the crisis, and found that there was an increase in the use of both antidepressants [9,26] and anxiolytics (benzodiazepines) [9] during the economic crisis. In fact, using a different design, Pérez-Romero et al. also found that there was an increase in the consumption of anxiolytics [21]. Barceló et al., point out that there was an increase in the severity, rather than the intensity, of mental health disorders in individuals who had already had disorders before the crisis. This increase occurred in those most likely to be unemployed, and the severity was accentuated in the toughest year of the economic crisis [9].

### 3.4. Time Bias

In terms of the number of studies that displayed time bias, our review found this to be the second most prevalent bias. It occurred mainly in studies that used time series, such as those in which the health outcome was ‘suicides’, ‘mortality’ and, to a lesser extent, ‘health inequalities’.

Time bias has two components. The first consists of the small number of periods after the onset of the crisis. None of the studies that analyzed these health outcomes took into consideration up to at least three years of post-crisis data [2] (in our case, 2014–2016).

With the exception of three (2 of 9 assessing suicides and 1 of 8 assessing mortality), the studies used data until 2011–2012 (that is, still in the middle of the Great Recession). They found that during the Great Recession, suicides remained stable or decreased, while mortality continued the downward trend observed before the Great Recession or, in any case, decreased more slowly than before the crisis.

There were, however, several exceptions. Using a different, although related, health outcome, Córdoba-Doña et al. found a sharp increase in suicide attempt rates in Andalusia (especially for adults aged 35–54 years old) after the onset of the Great Recession [40]. The effect the crisis had on this variable could be much more related to the recession than mortality from suicides was, as a consequence of a deterioration in mental health on account of unemployment (the unemployment rate in Andalusia in general, and in that age group in particular, was double the rest of Spain). Other exceptions were related to the cause of mortality and/or the population group analyzed. Llàcer et al. indicated an increase in mortality from intestinal infections, especially for adults over 75 years of age, and a change in the trend in mortality due to infections in the perinatal period [48]. Alonso et al. found that precisely alcohol-related mortality increased in the unemployed [45]. Benmarhnia et al. reported an increase in winter mortality for older adults (60 years of age or older) [46]. The remaining exceptions were related to the control of the unobserved confounding bias. In this sense, López-Bernal et al. [41] and Saurina et al. [44], when controlling temporal extra variability [33,34] and heterogeneity [56], found that the Great Recession was associated with an increase in suicides, particularly among males and those of working age (in Saurina et al., among working-aged women living in municipalities in Catalonia with 10,000 or more inhabitants [44]).

The second component of the time bias was the presence of potential lag effects. None of the studies contemplated their existence, despite the fact they could exist. Figure S1 in the supplement (Supplementary Figure S1) represents the crude mortality rate for Spain (per 1000 inhabitants) (source: Statistics National Institute, INE) (Figure S1). We placed the years 2009, 2012, and 2015 as vertical lines. Using, as most studies did, data up to 2011, it is observed that after the onset of the Great Recession, mortality continued to decrease. A change in trend can also be observed in 2010, although with data only until 2011, this trend cannot be assured as lasting. However, if more data were available, the change in trend would have been confirmed and two (relative) peaks would have been observed, one in 2012 (three years after the start of the first recession) and the other in 2015 (four years after the start of the second recession). There seems to be, therefore, a delayed effect that transpired after three or four years from the onset.

This effect, or at least that corresponding to the first recession, could only have been captured by the studies that have data until 2013–2014, although indirectly because it was not modelled explicitly. Furthermore, Alvárez-Gálvez et al. point out that the suicide rates were significantly reduced from 1995 to 2007, remained relatively stable from 2007 to 2011, and increased significantly from 2011 to 2014 [38]. Along the same lines, with data until 2013, Rivera et al. found that a decrease in economic growth and an increase in unemployment negatively affected suicide rates [43]. Meanwhile, mortality from cancer continued to decrease, although much more slowly, with the exceptions of colon cancer (in men) and lung cancer (in women), which continued to grow at the same rate as before the crisis [47].

### 3.5. Other Biases

As we have seen, most of the inconsistencies found in the studies that evaluated the effects of the Great Recession on practically all health outcomes can be summarized as having a lack of control of both observed and unobserved confounding.

While most of the studies evaluated as having low or moderate risk bias controlled for unobserved confounding (15 of 21, 71.4%), so too did some of those evaluated as having high risk. In this case, most control for a long-term temporal trend (although seasonal behavior is also controlled in one case) that could have cofounded the effects of the crisis on the health outcome. Somewhat fewer were studies that, by introducing random effects, control heterogeneity; that is, the variability in the health outcome not explained by the variable of interest (the crisis) or by the confounders.

Some of the studies evaluating the effects of the Great Recession on mortality and on suicides use statistical methods based on interrupted time series (also called intervention analysis in other contexts). These methods have been used to evaluate many types of interventions at a population level in a clearly defined time period. Some of those interventions evaluated were natural experiments. Natural experiment studies are observational designs in which an exogenous shock occurs that cannot be manipulated by the researcher. The Great Recession was, in fact, a natural experiment. It was an exogenous shock (to the health outcomes), to which a subpopulation was exposed (the population after the crisis), whereas it was not another (the population before the crisis). This allowed attributing changes in health outcomes to its exposure.

The problem is that, in some of these studies, the shock to which the population was exposed was not considered exogenous. In these, the onset of the crisis did not occur in the third quarter of 2008 (with annual data, in 2009) but was defined using endogenous criteria based on changes in the trend of crisis outcomes, either the health outcome itself or in unemployment. All of the studies considered that the crisis began in 2007. That the crisis began at one time or another has had important consequences on the results found. To illustrate this, we used the crude mortality data represented in Figure 1. Wrongly considering the onset of the Great Recession to have been in 2007 (the effects as of 2008), the average annual percent change (AAPC) in the crude mortality rate (per 1000 inhabitants) was −0.64% for the period 2001–2007 and −0.45% for the period 2008–2011. That is, the AAPC continued to decrease, although more slowly. However, considering that the onset of the crisis occurred in 2008 (the effects as of 2009), the AAPC for the period 2001–2008 was −0.69%, and 0.82% for the period 2009–2011. That is, there was a change in trend.

## 4. Discussion

In summary, we evaluated 60.38% of the studies reviewed (32 of 53) as having a high risk of bias (Table 3). This percentage was significantly lower than that found by Parmar et al. (73.00%, 30 of 41) [2]. The percentage of studies rated strong in the overall risk assessment was also much smaller (1.96% in our case vs. 5.00% in Parmar et al.). With the exception of ‘alcohol and illegal drugs’ and ‘health inequalities’ (in which we reviewed much fewer studies than for the rest of the health outcomes), we evaluated more than 50% of the studies in each health outcome as having a high risk of bias. We highlight ‘mortality’ in which we evaluated 87.5% of the studies as a high risk of bias.

The main biases that we found in our review were the evaluation problem, the time bias with its two components, an insufficient number of temporary periods after the crisis to obtain reliable estimators and not considering the existence of lag effects, the lack of control of the unobserved confounding, that is, lack of control of temporal trends and unobserved heterogeneity, and the non-exogeneity in the definition of the onset of the Great Recession. While the evaluation problem is associated with cross-sectional designs and, therefore, was more frequent in those health outcomes that use them (‘self-perceived health’, ‘mental health’, ‘use of and access to health services’, and ‘alcohol and illegal drugs’), the temporal bias and the non-exogeneity in the definition of the onset of the crisis are associated with longitudinal designs and were more frequent in health outcomes like ‘suicides’, ‘mortality’, and ‘health inequalities’. The lack of control of unobserved confounding occurred in virtually all health outcomes.

The studies we evaluated as having a low or moderate risk of bias (although also some that we evaluated as high risk) controlled those biases using a number of methods. In particular, in descending order in the number of studies, adjustment in the multivariate model of observed, above all, and unobserved confounding (in this case, mainly controlling the temporal trends and, to a lesser extent, the heterogeneity through random effects), stratification and matching. Many of the studies combined several of these methods.

The results from those studies that controlled the biases were quite consistent and in line with the results shown by other systematic reviews [2,66]. However, the results of the only two systematic reviews we were able to find for the case of Spain, only coincide with ours in terms of the effects of the crisis, in general, and the increase in unemployment, in particular, on mental health [38,39]. We believe that the differences could be explained by the high risk of bias we found in some of the studies reviewed.

The results of the studies that we reviewed and that we qualified as having a low or moderate risk of bias, found that the Great Recession increased the risk of declaring self-rated poor health. There was also a deterioration in mental health, both self-perceived and approximated by the increase of consumption of psychotropics. These declines in health (both, general and mental) occurred particularly among the most vulnerable groups of the population, such as the unemployed, as well as the groups with the greater risk of vulnerability, such as children.

Closely related to mental health, while alcohol-related disorders increased, the prevalence of hazardous drinking decreased (probably only in men), however, binge drinking, heavy smoking, and the use of illegal drugs increased.

Both the mortality rate and the suicide rate could have increased after the Great Recession, probably with a delay of three to four years. An increase in mortality was observed in specific causes of mortality (mortality by intestinal infections and mortality due to infections in the perinatal period) and/or in more vulnerable population groups (directly alcohol-related mortality among the unemployed, winter mortality in older adults). In the case of suicides, this increase would have occurred, above all, since the double-dip recession (i.e., 2011) and in the population most exposed to unemployment (males and population of both sexes of working age).

With regards to the use of health services, the onset of the Great Recession coincided with a decrease in general practitioner visits and hospitalizations and an increase in specialist visits.

## 5. Conclusions

We evaluated as having a moderate risk of bias only two studies (out of a total of seven) that analyzed other health outcomes [64,65]. Varea et al. show a significant increase in the prevalence of underweight babies at birth from 2008. They point out that this could be a combination of worsening socioeconomic conditions and increased psychosocial stress, even in the better-off strata [65]. Palència et al. find that the prevalence of small-for-gestational age increased following the onset of the crisis, and that a previous downward trend was interrupted [64].

This study may have several limitations. First of all, in the evaluation of the studies that we reviewed, there could have been some degree of subjectivity, particularly in a few of the domains. Secondly, we do not rule out the existence of a certain publication bias, especially in relation to those reports and publications not published in peer-reviewed journals. However, regarding the first limitation, it can be said that the evaluation criteria were quite objective and guided, while for the second, many of these reports have either already appeared as articles in peer-reviewed journals or their authors have already published part of them in these journals.

Finally, we could have overlooked a third mechanism (in addition to an increase in psychosocial stresses and a deterioration in social and economic conditions) by which the Great Recession had an impact on health, the influence of austerity measures on the healthcare system, in general, and on quality and availability of medical care, in particular. Thus, for example, from 2012 to 2018, the share of the public expenditures in gross domestic product decreased from 48% to 41%. However, as a consequence of the proximity of the implementation of these measures, there is very little evidence of the effects of such policy on the condition of a national health service system and none of the effects on health from this third mechanism. In addition, the little evidence does not have a simple interpretation. Thus, Lostao et al. indicate that after the restriction of universal health coverage in Spain, subjects with lower incomes had a higher frequency of physician consultations [67]. In the same vein, Córdoba-Doña et al. point out that, for Andalusia, Spain, despite the cuts in welfare benefits and health services, provision during the early years of the recession, inequalities in healthcare utilization largely remained in favor of the less well-off [57]. The answer could be in the findings of Palladino et al., who using data from the Survey of Health, Aging, and Retirement in Europe in 11 European countries, including Spain, find that poorer populations were less likely than those in the highest income quintile to incur out-of-pocket expenditure and reported lower out-of-pocket expenditures [68].

## Key points

We evaluated 60.38% of the studies reviewed (32 of 53) as having a high risk of bias.

The evaluation problem, the time bias and the non-exogeneity in the definition of the onset of the Great Recession are the main biases we found in our review.

The Great Recession increased the risk of declaring poor self-rated health and the deterioration of mental health.

Both the mortality rate and the suicide rate may well have increased after the Great Recession, probably after a three–four years delay.

## Figures and Tables

**Table 1:** Bias tool assessment.

Bias Domain ^1^	Question to Consider	Indicator	Score (1: Low Risk of Bias; 2: Medium Risk; 3: High risk)
1. Confounding	Did the study analysis adjust potential confounders appropriately?	Confounders adjusted	1: most confounders
			2: some confounders
			3: none or cannot tell
2. Measurement error in health outcome	What was the heterogeneity of indicators used in the study?	Health outcome	1: clinical indicator ^2^
			2: self-reported validated tool
			3: self-reported tool but not clear if it had been validated ^2^
3. Measurement error in exposure variable	Did the study consider macroeconomic variables in the analysis?	Number of macroeconomic variables	1: >1 variable
			2: 1 variable
			3: none
4. Ecological fallacy	Were inferences about individuals deduced from inference for the group to which those individuals belonged?	Study design	1: non-ecological
			3: ecological
5. Time bias	Were there sufficiently long numbers of time periods in order to obtain reliable estimates?	Factors to be considered: >10 years of data; ≥3 years of data post crisis: potential lag effects	1: 3 factors were considered
			2: ≥2
			3: one or none
6. Evaluation problem	Was an event (e.g., the Great Recession) evaluated using data in a different form from a cross section (e.g., health surveys) before and after that event?	Number of methods used to control bias	1: >1 method
			2: 1 method
			3: none
7. Unobserved confounding	Did the study control the heterogeneity (i.e., unobserved confounding) and temporal extra variability?	Number of methods used to control bias	1: >1 method
			2: 1 method
			3: none
Study Overall Rating	Strong (low risk of bias): none of the domains is rated as 3		
	Moderate: up to 2 domains were rated as 3		
	Weak (high risk of bias): ≥3 domains were rated as 3		

^1^ Domains 1 to 6 are from Parmar et al. [2], while domains 7 and 8 were added specifically for this study. ^2^ Or officially recorded data that are not likely to be misreported or misstated.

**Table 2:** Evaluation of bias for the 53 studies in the systematic review.

	Selection	Evaluation	Ecological Fallacy	Confounding	Unobserved Confounding	Time	Measurement Error		Number of 3 (High Risk)	Overall Rating
							Exposure	Outcome		
**Mental Health**										
Aguilar-Palacio, 2015 [6]	1	3	1	2	3	3	2	2	3	3
Arroyo-Borrell, 2017 [7]	1	2	1	1	1	3	3	2	2	2
Bacigalupe, 2016 [8]	1	3	1	2	3	1	3	2	3	3
Barceló, 2016 [9]	1	1	1	1	1	1	2	1	0	1
Bartoll, 2014 [10]	1	3	1	2	3	3	3	2	4	3
Basterra, 2017 [11]	1	3	1	2	3	3	3	2	4	3
Córdoba-Doña, 2016 [12]	1	3	1	1	3	3	3	2	4	3
Fernández-García, 2018 [13]	1	1	3	3	3	2	3	1	4	3
Gili, 2013 [14]	1	2	1	2	3	3	1	2	2	2
Gili, 2014 [15]	1	1	3	3	3	1	3	1	4	3
Gili, 2016 [16]	1	3	1	2	3	3	1	2	3	3
Gotsens, 2015 [17]	2	3	1	2	3	3	1	2	3	3
Iglesias-García, 2014 [18]	2	1	1	3	3	1	2	2	2	2
Medel-Herreros, 2017 [19]	1	1	3	3	3	1	3	1	4	3
Navarro-Mateu, 2015 [20]	2	3	1	1	3	3	3	2	4	3
Pérez-Romero, 2016 [21]	2	3	1	1	3	3	3	2	4	3
Rajmil, 2013 [22]	1	3	1	2	3	3	2	2	3	3
Rajmil, 2015 [23]	1	3	1	3	3	2	1	2	3	3
Robert, 2014 [24]	3	3	1	1	3	3	3	2	5	3
Ruíz-Pérez, 2017 [25]	1	2	3	1	1	3	1	2	2	2
Sicras-Mainar, 2015 [26]	1	1	1	3	3	3	3	1	4	3
Utzet, 2016 [27]	2	3	1	3	3	3	3	2	5	3
Zapata-Moya, 2015 [28]	1	2	1	2	1	2	3	2	2	2
**Self-Perceived Health**										
Aguilar-Palacio, 2015 [5]	1	3	1	2	3	3	2	2	3	3
Arroyo-Borrell, 2015 [29]	1	1	1	1	1	3	3	2	2	2
Barroso, 2016 [30]	2	3	1	1	3	3	3	2	4	3
Bartoll, 2015 [31]	1	3	1	2	3	2	2	2	2	2
Calzón-Fernández, 2017 [32]	2	3	1	1	3	3	3	2	3	3
Fornell, 2018 [33]	1	2	1	2	1	2	3	2	2	2
Gotsens, 2015 [17]	2	3	1	2	3	3	1	2	3	3
López-del-Amo, 2018 [34]	1	2	1	1	1	3	1	2	1	2
Pérez-Romero, 2016 [21]	2	2	1	1	3	3	3	2	3	3
Rajmil, 2013 [22]	1	3	1	2	3	3	2	2	3	3
Rajmil, 2015 [23]	1	2	1	3	3	2	1	2	2	2
Regidor, 2014 [35]	1	3	3	3	3	1	3	1	4	3
Urbanos-Garrido, 2015 [36]	1	1	1	1	3	3	3	2	3	3
Vásquez-Vera, 2016 [37]	3	2	1	1	3	3	3	2	4	3
Zapata-Moya, 2015 [28]	1	2	1	2	1	2	3	2	2	2
**Suicides**										
Álvarez-Gálvez, 2017 [38]	1	1	3	2	3	1	1	1	2	2
Borrell, 2017 [39]	1	1	3	2	3	1	3	1	3	3
Córdoba-Doña, 2014 [40]	1	1	3	2	2	2	2	1	1	2
Gili, 2014 [15]	1	1	3	3	3	1	3	1	4	3
López-Bernal, 2013 [41]	2	1	3	2	2	3	3	1	3	3
Miret, 2014 [42]	3	3	1	2	3	3	3	2	5	3
Rivera, 2016 [43]	1	2	3	2	1	1	1	1	1	2
Ruíz-Pérez, 2017 [25]	1	1	3	3	3	1	3	1	4	3
Saurina, 2015 [44]	1	1	3	3	1	1	2	1	2	2
**Mortality**										
Alonso 2017 [45]	1	1	1	3	3	2	3	1	3	3
Benmarhnia, 2014 [46]	1	1	3	3	2	2	3	1	3	3
Ferrando, 2018 [47]	1	1	3	3	1	2	3	1	3	3
Llàcer, 2014 [48]	2	1	1	3	3	1	3	1	3	3
Maynou, 2016 [49]	1	1	3	2	1	3	2	1	2	2
Regidor, 2014 [35]	1	1	3	3	3	1	3	1	4	3
Regidor 2016 [50]	1	1	1	3	3	2	3	1	3	3
Ruíz-Ramos, 2014 [51]	1	1	3	3	2	2	3	1	3	3
**Alcohol and Illegal Drugs**										
Bosque-Prous, 2017 [52]	1	3	1	1	3	3	3	2	4	3
Collell, 2015 [53]	2	3	1	1	3	3	3	2	4	3
Gili, 2013 [14]	1	2	1	2	3	3	1	2	2	2
Martín-Bassols, 2016 [54]	2	3	1	1	2	3	2	2	2	2
**Health Inequalities**										
Abásolo, 2017 [55]	1	2	1	1	1	3	3	2	2	2
Coveney, 2016 [56]	1	1	1	1	2	1	3	2	1	2
Maynou, 2016 [49]	1	1	3	2	1	3	2	1	2	2
**Use of and Access to Health Services**										
Abásolo, 2017 [55]	1	2	1	1	1	3	3	2	2	2
Córdoba-Doña, 2018 [57]	1	3	1	1	3	3	3	2	4	3
García-Subirats, 2014 [58]	1	3	1	1	3	3	3	2	4	3
Lostao, 2017 [59]	1	3	1	2	3	3	3	2	4	3
**Other**										
Calzón-Fernández, 2015 [60]	1	3	1	1	3	3	3	2	4	3
Fernández, 2015 [61]	3	1	1	1	3	3	3	2	4	3
Llàcer, 2014 [48]	2	1	1	3	3	1	3	1	3	3
Larrañaga, 2014 [62]	2	3	1	3	3	1	3	2	4	3
Lorenzo-Carrascosa,2016 [63]	1	1	3	3	3	1	3	1	4	3
Palència, 2018 [64]	1	1	1	2	1	2	3	1	1	2
Varea, 2016 [65]	1	1	1	2	3	1	1	1	1	2

**Table 3:** Studies with high risk of bias (rated with 3) by health outcome and bias domain.

Health Outcome		Selection	Evaluation	Ecological Fallacy	Confounding	Unobserved Confounding	Time	Measurement Error Exposure	Measurement Error Outcome	Overall Rating	Total
Mental health	n	1	13	4	7	19	15	14	0	17	23
	%	4.3%	56.5%	17.4%	30.4%	82.6%	65.2%	60.9%	0.0%	73.9%	32.4%
Self-perceived health	n	1	7	1	2	11	10	8	0	9	14
	%	7.1%	50.0%	7.1%	14.3%	78.6%	71.4%	57.1%	0.0%	64.3%	19.7%
Suicides	n	1	1	8	3	5	2	5	0	5	9
	%	11.1%	11.1%	88.9%	33.3%	55.6%	22.2%	55.6%	0.0%	55.6%	12.7%
Mortality	n	0	0	5	7	4	1	7	0	7	8
	%	0.0%	0.0%	62.5%	87.5%	50.0%	12.5%	87.5%	0.0%	87.5%	11.3%
Alcohol and illegal drugs	n	0	3	0	0	3	4	2	0	2	4
	%	0.0%	75.0%	0.0%	0.0%	75.0%	100.0%	50.0%	0.0%	50.0%	5.6%
Health inequalities	n	0	0	1	0	0	2	2	0	0	3
	%	0.0%	0.0%	33.3%	0.0%	0.0%	66.7%	66.7%	0.0%	0.0%	4.2%
Use and access of health services	n	0	2	0	0	2	3	3	0	2	3
	%	0.0%	66.7%	0.0%	0.0%	66.7%	100.0%	100.0%	0.0%	66.7%	4.2%
Other	n	1	2	1	3	6	2	6	0	5	7
	%	14.3%	28.6%	14.3%	42.9%	85.7%	28.6%	85.7%	0.0%	71.4%	9.9%
Total	n	4	28	20	22	50	39	47	0	47	71
	%	5.6%	39.4%	28.2%	31.0%	70.4%	54.9%	66.2%	0.0%	66.2%	100.0%

Number and percentage of studies with high risk of bias (i.e., rated with 3).

## Supplementary Materials

The following are available online at https://www.mdpi.com/1660-4601/16/14/2479/s1, Figure S1: Crude mortality rate for Spain (per 1000 inhabitants), Table S1: Studies include in the systematic revision.

## Data sharing statement

All the studies as well as the data and the code used to create Figure 1 can be requested from the corresponding author (marc.saez@udg.edu).
