# Supplementary material for: Evaluation of the Biases in the Studies that Assess the Effects of the Great Recession on Health. A Systematic Review
Source: Int J Environ Res Public Health. 2019 Jul 11;16(14):2479. doi: 10.3390/ijerph16142479 (PMC6678595; doi:10.3390/ijerph16142479)
Supplement: Supplementary file 1 [file ijerph-16-02479-s001.zip › Table S1.docx]

**Table S1. Studies include in the systematic revision**

Mental health

Aguilar-Palacio I, Carrera-Lasfuentes P, Rabanaque MJ. Youth unemployment and economic recession in Spain: influence on health and lifestyles in young people (16-24 years old). *Int J Public Health* 2015; 60:427-435.

Arroyo-Borrell E, Renart G, Saurina C, Saez M. Influence maternal background has on children’s mental health. *Int J Equity Health.* 2017; 16(1):63.

Bacigalupe A, Esnaola S, Martín U. The impact of the Great Recession on mental health and its inequalities: the case of a Southern European region, 1997-2013. *Int J Equity Health.* 2016; 15:17.

Barceló MA, Coll-Negre M, Coll-de-Tuero G, Saez M. Effects of the financial crisis on psychotropic drug consumption in a cohort from a semi-urban region in Catalonia, Spain. *PloS One.* 2016; 11(2):e0148594.

Bartoll X, Pale L, Malmusi D, Suhrcke M, Borrell C. The evolution of mental health in Spain during the economic crisis. *Eur J Public Health* 2014; 24(3):415-418.

Basterra V. Prevalence trends of high risk of mental disorders in the Spanish adult population: 2006-2012 [in Spanish]. *Gac Sanit.* 2017; 31(4):324-326.

Córdoba-Doña JA, Escolar-Pujolar A, San Sebastián M, Gustafsson PE. How are the employed and unemployed affected by the economic crisis in Spain? Educational inequalities, life conditions and mental health in a context of high unemployment. *BMC Public Health* 2016; 16:267.

Fernández-García MA, Olry-de-Labry-Lima A, Ferrer-López I, Bermúdez-Tamayo C. Analysis of changes in trends in the consumption rates of benzodiazepines and benzodiazepine-related drugs. *J Pharm Policy Pract.* 2018; 11:1.

Gili M, Roca M, Basu S, McKee M, Stuckler D. The mental health risks of economic crisis in Spain: evidence from primary care centres, 2006 and 2010. *Eur J Public Health.* 2013; 23(1):103-108.

Gili M, García-Campayo J, Roca M. Economic crisis and mental health. SESPAS Report 2014 [in Spanish]. *Gac Sanit.* 2014; Suppl 1:104-108.

Gili M, López-Navarro E, Castro A, Homar C, Navarro C, García-Toro M, García-Campayo J, Roca M. Gender differences in mental health during the economic crisis. *Psicothema.* 2016; 28(4):407-413.

Gotsens M, Malmusi D, Villarroel N, Vives-Cases C, García-Subirats I, Hernando C, Borrell C. Health inequality between immigrants and natives in Spain: the loss of the healthy immigrant effect in times of economic crisis. *Eur J Public Health* 2015;25:923-929.

Iglesias-García C, Sáiz-Martínez P, García-Portilla MP, Bousoño-García M, Jiménez-Treviño L, Sánchez-Lasheras F, Bobes J. Effects of the economic crisis on demand due to mental disorders in Asturias: data from the Asturias Cumulative Psychiatric Case Register (2000-2010). *Actas Esp Psiquiatr.* 2014; 42(3):108-115.

Medel-Herrero A, Gómez-Beneyto M. The impact of the 2008 economic crisis on the increasing number of psychiatric inpatients. *Rev Psiquiatr Salud Ment.* 2017; S1888-9891(17ç920126-X [Epub ahead of print].

Navarro-Mateu F, Tormo MJ, Salmerón D, Vilagut G, Navarro C, Ruíz-Merino G, Escámez T, Júdez J, Martínez S, Kessler RC, Alonso J. Prevalence of mental disorders in the South-East of Spain, one of the European regions most affected by the economic crisis: The cross-sectional PEGASUS-Murcia Project. *PLos One.* 2015; 10(9):e0137293.

Pérez-Romero S, Gascón-Cánovas JJ, de la Cruz-Sánchez E. Sánchez-Ruíz JF, Parra-Hidalgo P, Monteagudo-Piqueras O. Economic recession (2006-2012) and changes in the health status of the Spanish population [in Spanish]. *Salud Publica Mex* 2016; 58(1):41-48.

Rajmil L, Medina-Bustos A, Fernández de Sanmamed MJ, Mompart-Penina A. Impact of the economic crisis on children’s health in Catalonia: a before-after approach. *BMJ Open* 2013; 3:e003286.

Rajmil L, Siddigi A, Taylor-Robinson D, Spencer N. Understanding the impact of the economic crisis on child health: the case of Spain. *Int J Equity Health* 2015; 14:95.

Robert G, Martínez JM, García AM, Benavides FG, Ronda E. From the boom to the crisis: changes in employment conditions of immigrants in Spain and their effects on mental health. *Eur J Public Health.* 2014; 24(3):404-409.

Ruíz-Pérez I, Rodríguez-Barranco M, Rojas-García A, Mendoza-García O. Economic crisis and suicides in Spain. Socio-demographic and regional variability. *Eur J Health Econ.* 2017; 18(3):313-320.

Sicras-Mainar A, Navarro-Artieda R. Use of antidepressants in the treatment of major depressive disorder in primary care during a period of economic crisis. *Neuropsychiatr Dis Treat.*  2015. 12:29-40.

Utzet M, Navarro A, Llorens C, Muntaner C, Moncada S. Is the worsening of psychosocial exposures associated with mental health? Comparing two population-based cross-sectional studies in Spain, 2005-2010. *Am J Ind Med.* 2016; 59(5):399-407.

Zapata-Moya AR, Buffel V, Navarro-Yáñez CJ, Bracke P. Social inequality in morbidity, framed within the current economic crisis in Spain. *Int J Equity Health.* 2015; 14:131.

Self-perceived health

Aguilar-Palacio I, Carrera-Lasfuentes P, Rabanaque MJ. Youth unemployment and economic recession in Spain: influence on health and lifestyles in young people (16-24 years old). *Int J Public Health* 2015; 60:427-435.

Arroyo E, Renart G, Saez M. How the economic recession has changed the likelihood of reporting poor self-rated health in Spain. *Int J Equity Health* 2015; 14:149.

Barroso C, Abásolo I, Cáceres JJ. Health inequalities by socioeconomic characteristics in Spain: the economic crisis effect. *Int J Equity Health.*  2016; 15:62.

Bartoll X, Toffolutti V, Malmusi D, Palència L, Borrell C, Suhrcke M. Health and health behaviours before and during the Great Recession, overall and by socioeconomic status, using data from four repeated cross-sectional health surveys in Spain (2001-2012). *BMC Public Health* 2015;15:865.

Calzón-Fernández S, Fernández-Ajuria A, López-del-Amo-González MP, Martín-Martín JJ. Sex differences of perceived health before and during the economic crisis (2007 and 2011). Spain [in Spanish]. *Rev Esp Salud Publica* 2017; 16:91.

Fornell B, Correa M, López del Amo MP, Martín JJ. Influence of changes in the Spanish labor market during the economic crisis (2007-2011) on perceived health. *Qual Life Res.* 2018; doi: 10.1007/s11136-018-1824-5 (in press).

Gotsens M, Malmusi D, Villarroel N, Vives-Cases C, García-Subirats I, Hernando C, Borrell C. Health inequality between immigrants and natives in Spain: the loss of the healthy immigrant effect in times of economic crisis. *Eur J Public Health* 2015;25:923-929.

López-del-Amo MP, Benítez V, Martín-Martín JJ. Long term unemployment, income, poverty, and social public expenditure, and their relationship with self-perceived health in Spain (2007-2011). *BMC Public Health* 2018; 18(1):133.

Pérez-Romero S, Gascón-Cánovas JJ, de la Cruz-Sánchez E. Sánchez-Ruíz JF, Parra-Hidalgo P, Monteagudo-Piqueras O. Economic recession (2006-2012) and changes in the health status of the Spanish population [in Spanish]. *Salud Publica Mex* 2016; 58(1):41-48.

Rajmil L, Medina-Bustos A, Fernández de Sanmamed MJ, Mompart-Penina A. Impact of the economic crisis on children’s health in Catalonia: a before-after approach. *BMJ Open* 2013; 3:e003286.

Rajmil L, Siddigi A, Taylor-Robinson D, Spencer N. Understanding the impact of the economic crisis on child health: the case of Spain. *Int J Equity Health* 2015; 14:95.

Regidor E, Barrio G, Bravo MJ, de la Fuente L. Has health in Spain been declining since the economic crisis? *J Epidemiol Community Health* 2014;68:280-282.

Urbanos-Garrido RM, López-Valcárcel BG. The influence of the economic crisis on the association between unemployment and health: an empirical analysis for Spain. *Eur J Health Econ.*  2015; 16(2):175-184.

Vásquez-Vera H, Rodríguez-Sanz M, Palència L, Borrell C. Foreclosure and health in Southern Europe: Results from the platform for people affected by mortgages. *J Urban Health* 2016; 93(2):312-330.

Zapata-Moya AR, Buffel V, Navarro-Yáñez CJ, Bracke P. Social inequality in morbidity, framed within the current economic crisis in Spain. *Int J Equity Health.* 2015; 14:131.

Suicides

Álvarez-Gálvez J, Salinas-Pérez JA, Rodero-Cosano ML, Salvador-Carulla L. Methodological barriers to studying the association between the economic crisis and suicide in Spain. *BMC Public Health.* 2017; 17(1):694.

Borrell C, Marí-Dell’Olmo M, Gotsens M, Calvo M, Rodríguez-Sanz M, Bartoll X, Esnaola S. Socioeconomic inequalities in suicide mortality before and after the economic recession in Spain. *BMC Public Health* 2017; 17(1):772.

Córdoba-Doña JA, San Sebastián M, Escolar-Pujolar A, Martínez-Faure JE, Gustafsson PE. Economic crisis and suicidal behaviour: the role of unemployment, sex and age in Andalusia, southern Spain. *Int J Equity Health.* 2014; 13:55.

Gili M, García-Campayo J, Roca M. Economic crisis and mental health. SESPAS Report 2014 [in Spanish]. *Gac Sanit.* 2014; Suppl 1:104-108.

López-Bernal JA, Gasparrini A, Artundo CM, McKee M. The effect of the late 2000s financial crisis on suicides in Spain: an interrupted time-series analysis. *Eur J Public Health.* 2013; 23(5):732-736.

Miret M, Caballero FF, Huerta-Ramírez R, Moneta MV, Olaya B, Chatterji S, Haro JM, Ayuso-Mateos JL. Factors associated with suicidal ideation and attempts in Spain for different age groups. Prevalence before and after the onset of the economic crisis. *J Affect Disord.* 2014; 163:1-9.

Rivera B, Casal B, Currais L. Crisis, suicide and labour productivity losses in Spain. *Eur J Health Econ* 2017; 18(1):83-96.

Ruíz-Pérez I, Rodríguez-Barranco M, Rojas-García A, Mendoza-García O. Economic crisis and suicides in Spain. Socio-demographic and regional variability. *Eur J Health Econ.* 2017; 18(3):313-320.

Saurina C, Marzo M, Saez M. Inequalities in suicide mortality rates and the economic recession in the municipalities of Catalonia, Spain. *Int J Equity Health.* 2015; 14:75.

Mortality

Alonso I, Vallejo F, Regidor E, Belza MJ, Sordo L, Otero-García L, Barrio G. Changes in directly alcohol-attributable mortality during the great recession by employment status in Spain: a population cohort of 22 million people. *J Epidemiol Community Health* 2017; 71(8):736-744.

Benmarhnia T, Zunzunegui MV, Llàcer A, Béland F. Impact of the economic crisis on the health of older persons in Spain: research clues based on an analysis of mortality. SESPAS Report 2014. *Gac Sanit.* 2014; Suppl 1:137-141.

Ferrando J, Palència L, Gotsens M, Puig-Barrachina V, Marí-Dell’Olmo M, Rodríguez-Sanz M, Bartoll X, Borrell C. Trends in cancer mortality in Spain: the influence of the financial crisis. *Gac Sanit.* 2018; pii: S0213-9111(18)30005-0.

Llàcer A, Fernández-Cuenca R, Martínez-Navarro F. Economic crisis and communicable diseases. SESPAS Report 2014. [in Spanish]. *Gac Sanit.* 2014; Suppl 1:97-103.

Maynou L, Saez M, López-Casasnovas G. Has the economic crisis widened the intraurban socioeconomic inequalities in mortality? The case of Barcelona, Spain. *J Epidemiol Community Health.* 2016; 70(2):114-124.

Regidor E, Barrio G, Bravo MJ, de la Fuente L. Has health in Spain been declining since the economic crisis? *J Epidemiol Community Health* 2014;68:280-282.

Regidor E, Vallejo F, Granados JAT, Viciana-Fernández FJ, de la Fuente L, Barrio G. Mortality decrease according to socioeconomic groups during the economic crisis in Spain: a cohort study of 36 million people. *Lancet.* 2016; 388(10060):2642-2652.

Ruíz-Ramos M, Córdoba-Donña JA, Bacigalupe A, Juárez S, Escolar-Pujolar A. The economic crisis at the beginning of the XXI century and mortality in Spain. Trend and impact on social inequalities. SESPAS Report 2014. [in Spanish]. *Gac Sanit.* 2014; Suppl 1:89-96.

Alcohol and illegal drugs

Bosque-Prous M, Kunst AE, Brugal MT, Espelt A. Changes in alcohol consumption in the 50- to 64-year-old European economically active population during an economic crisis. *Eur J Public Health.* 2017; 27(4):711-716.

Collell E, Sánchez-Niubò A, Delclós GL, Benavides FG, Domingo-Salvany A. Economic crisis and changes in drug use in the Spanish economically active population. *Addiction.* 2015; 110(7):1129-1137.

Gili M, Roca M, Basu S, McKee M, Stuckler D. The mental health risks of economic crisis in Spain: evidence from primary care centres, 2006 and 2010. *Eur J Public Health.* 2013; 23(1):103-108.

Martin-Bassols N, Vall-Castelló J. Effects of the great recession on drugs consumption in Spain. *Econ Hum Biol.* 2016; 22:103-116.

Health inequalities

Abásolo I, Saez M, López-Casasnovas G. Financial crisis and income-related inequalities in the universal provision of a public service: the case of healthcare in Spain. *Int J Equity Health.* 2017; 16(1):134.

Coveney M, García-Gómez P, Van Doorslaer E, Van Ourti T. Health disparities by income in Spain before and after the economic crisis. *Health Econ.* 2016; Suppl 2:141-158.

Maynou L, Saez M, López-Casasnovas G. Has the economic crisis widened the intraurban socioeconomic inequalities in mortality? The case of Barcelona, Spain. *J Epidemiol Community Health.* 2016; 70(2):114-124.

Use and access of health services

Abásolo I, Saez M, López-Casasnovas G. Financial crisis and income-related inequalities in the universal provision of a public service: the case of healthcare in Spain. *Int J Equity Health.* 2017; 16(1):134.

Córdoba-Doña JA, Escolar-Pujolar A, San Sebastián M, Gustafsson PE. Withstanding austerity: Equity in health services utilisation in the first stage of the economic recession in Southern Spain. *PloS One* 2018; 13(3):e0195293.

García-Subirats I, Vargas I, Sanz B, Malmusi D, Ronda E, Ballesta M, Luisa Vázquez M. Changes in access to health services of the immigrant and native-born population in Spain in the context of economic crisis. *Int J Environ Res Public Health.* 2014; 11(10):10182-10201.

Lostao L, Geyer S, Albaladejo R, Moreno-Lostao A, Santos JM, Regidor E. Socioeconomic position and health services use in Germany and Spain during the Great Recession. *PLoS One.* 2017; 12(8):e0183325.

Other

Calzón-Fernández S, Fernández-Ajuría A, Martín JJ, Murphy MJ. The impact of the economic crisis on unmet dental care needs in Spain. *J Epidemiol Community Health.* 2015; 69(9):880-885.

Fernández A, García-Alonso J, Royo-Pastor C, Garrell-Corbera I, Rengel-Chica J, Agudo-Ugena J, Ramos A, Mendive J. Effects of the economic crisis and social support on health-related quality of life: first wave of a longitudinal study in Spain. *Br J Gen Pract.* 2015; 65(632):e198-e203.

Llàcer A, Fernández-Cuenca R, Martínez-Navarro F. Economic crisis and communicable diseases. SESPAS Report 2014. [in Spanish]. *Gac Sanit.* 2014; Suppl 1:97-103.

Larrañaga I, Martín U, Bacigalupe A. Sexual and reproductive health and the economic crisis in Spain. SESPAS report 2014. *Gac Sanit.* 2014; 28(S1):109-115.

Lorenzo-Carrascosa L. The health of the elderly in Spain during the economic crisis [in Spanish]. *Rev Esp Geriatr Gerontol.* 2016; 53(1):12-14.

Palència L, Puig-Barrachina V, Marí-Dell’Olmo M, Gotsens M, Rodríguez-Sanz M, Bartoll X, Pérez G, IMCRISES members. Trends in small-for-gestational age before and after the economic crisis in Spain. *Eur J Public Health* 2018; 28(2):325-327.

Varea C, Terán JM, Bernís B, Bogin B, González-González A. Is the economic crisis affecting birth outcome in Spain? Evaluation of temporal trend in underweight at birth (2003-2012). *Ann Hum Biol.* 2016; 43(2):169-182.
