# Supplementary material for: Evaluation of the Biases in the Studies that Assess the Effects of the Great Recession on Health. A Systematic Review
Source: Int J Environ Res Public Health. 2019 Jul 11;16(14):2479. doi: 10.3390/ijerph16142479 (PMC6678595; doi:10.3390/ijerph16142479)
Supplement: Supplementary file 1 [file ijerph-16-02479-s001.zip › Figure S1.docx]

**Figure S1.- Crude mortality rate for Spain (per 1000 inhabitants)**


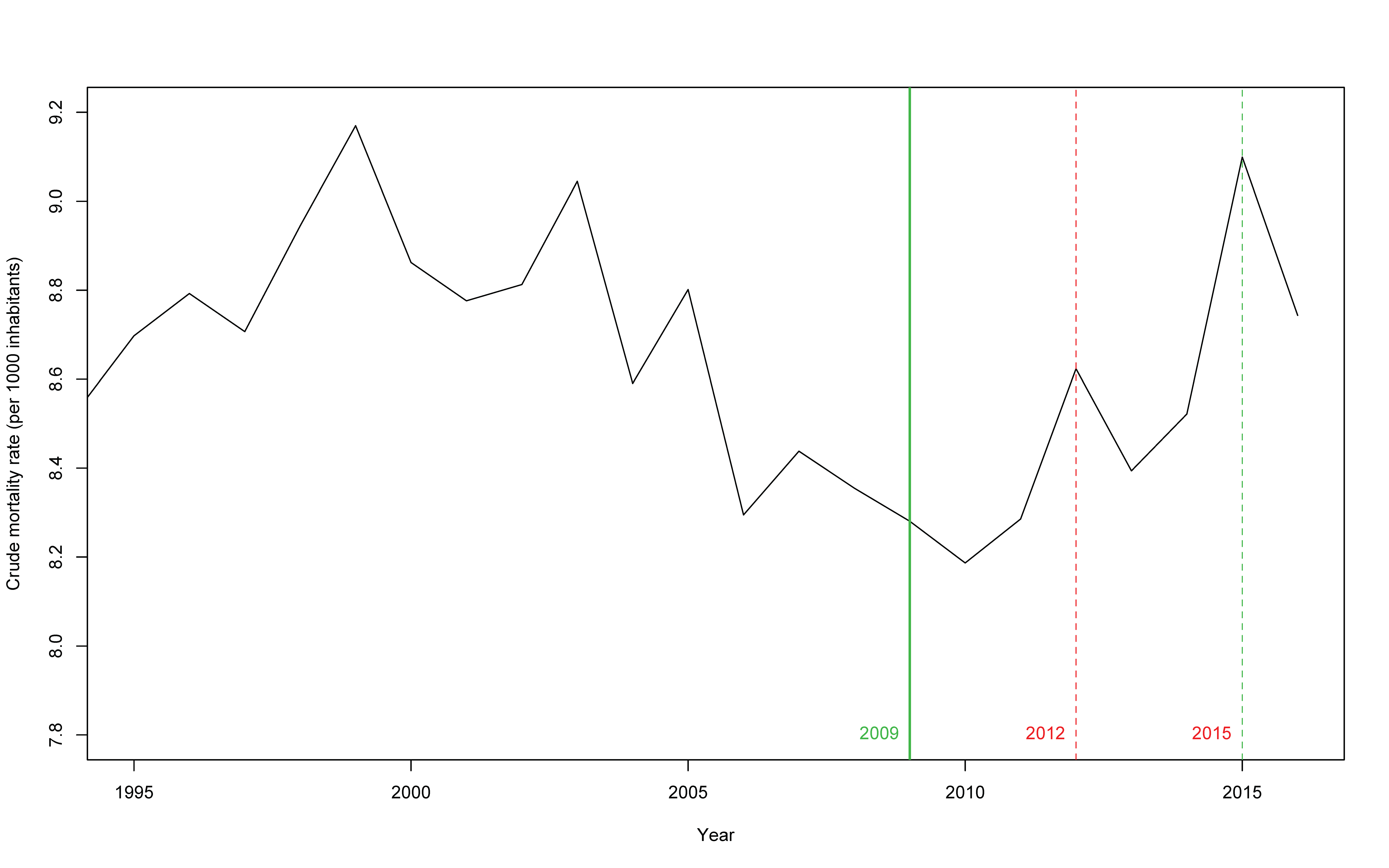


Source: Statistics National Institute, INE
